# Effects of Different Training Intensity Distributions Between Elite Cross-Country Skiers and Nordic-Combined Athletes During Live High-Train Low

**DOI:** 10.3389/fphys.2018.00932

**Published:** 2018-07-19

**Authors:** Laurent Schmitt, Sarah J. Willis, Nicolas Coulmy, Gregoire P. Millet

**Affiliations:** ^1^National School of Mountain Sports/National Ski-Nordic Centre, Premanon, France; ^2^Institute of Sport Sciences, Faculty of Biology and Medicine, University of Lausanne, Lausanne, Switzerland; ^3^French Ski Federation, Annecy, France

**Keywords:** altitude training, performance, Nordic-ski, fatigue, heart rate variability

## Abstract

**Purpose:** To analyze the effects of different training strategies (i.e., mainly intensity distribution) during living high – training low (LHTL) between elite cross-country skiers and Nordic-combined athletes.

**Methods:** 12 cross-country skiers (XC) (7 men, 5 women), and 8 male Nordic combined (NC) of the French national teams were monitored during 15 days of LHTL. The distribution of training at low-intensity (LIT), below the first ventilatory threshold (VT1), was 80% and 55% in XC and NC respectively. Daily, they filled a questionnaire of fatigue, and performed a heart rate variability (HRV) test. Prior (Pre) and immediately after (Post), athletes performed a treadmill incremental running test for determination of $\dot{\text{V}}$O_2max_ and $\dot{\text{V}}$O_2_ at the second ventilatory threshold ($\dot{\text{V}}$O_2V T2_), a field roller-skiing test with blood lactate ([La-]) assessment.

**Results:** The training volume was in XC and NC, respectively: at LIT: 45.9 ± 6.4 vs. 23.9 ± 2.8 h (*p* < 0.001), at moderate intensity: 1.9 ± 0.5 vs. 3.0 ± 0.4 h, (*p* < 0.001), at high intensity: 1.2 ± 0.9 vs. 1.4 ± 02 h (*p* = 0.05), in strength (and jump in NC): 7.1 ± 1.5 vs. 18.4 ± 2.7 h, (*p* < 0.001). Field roller-skiing performance was improved (-2.9 ± 1.6%, *p* < 0.001) in XC but decreased (4.1 ± 2.6%, *p* < 0.01) in NC. [La-] was unchanged (-4.1 ± 14.2%, *p* = 0.3) in XC but decreased (-27.0 ± 11.1%, *p* < 0.001) in NC. Changes in field roller-skiing performance and in [La-] were correlated (*r* = -0.77, *p* < 0.001). $\dot{\text{V}}$O_2max_ increased in both XC and NC (3.7 ± 4.2%, *p* = 0.01 vs. 3.7 ± 2.2%, *p* = 0.002) but $\dot{\text{V}}$O_2V T2_ increased only in XC (7.3 ± 5.8%, *p* = 0.002). HRV analysis showed differences between XC and NC mainly in high spectral frequency in the supine position (HF_SU_). All NC skiers showed some signs of overreaching at Post.

**Conclusion:** During LHTL, despite a higher training volume, XC improved specific performance and aerobic capacities, while NC did not. All NC skiers showed fatigue states. These findings suggest that a large amount of LIT with a moderate volume of strength and speed training is required during LHTL in endurance athletes.

## Introduction

Live high train low (LHTL) is a hypoxic training method used by many endurance athletes (Stray-Gundersen and Levine, 1999). If correctly prescribed and monitored, LHTL has been shown to be effective for endurance athletes, including those with an initial high aerobic fitness or hemoglobin mass (Millet et al., 2017). Based on 20 years of research since the original study in Levine and Stray-Gundersen (1997), LHTL is known for inducing a 1–3% larger improvement in specific endurance performance, when compared to similar normoxic training (Bonetti and Hopkins, 2009; Millet et al., 2010). However, the responses to altitude training remain complex since hypoxia and training stresses are combined (Schmitt et al., 2006). In hypoxia, the reduced inspired pressure of oxygen (PiO_2_) represents an additional stress, which has been shown to alter the autonomous regulation of the nervous system and subsequently to modify responses in heart rate variability (HRV) (Schmitt et al., 2006) with a combination of increased sympathetic and decreased parasympathetic nervous activities (Sevre et al., 2001; Schmitt et al., 2006). Schmitt et al. (2006, 2008) have shown that HRV parameters are modified differently depending on the intensity of training performed in altitude. Specifically, the combination of moderate altitude (<3000 m) and low intensity training (LIT), below the first ventilatory threshold (VT1), has been shown to be quite favorable to the development of aerobic capacities (Schmitt et al., 2006, 2008). Conversely, high-intensity exercises and the training loads (TL) have to be reduced, particularly during the first 7–10 days of an altitude training camp (Millet et al., 2010). In the case that the recovery capacities of an athlete are compromised, the athlete enters an overreached state. In this case, HRV parameters help diagnosing different fatigue states (Schmitt et al., 2013, 2015).

It is of practical interest to compare different groups of athletes performing LHTL. *Cross-country (XC) skiers* and *Nordic-combined (NC) athletes* have different characteristics and training contents as reported in Norwegian elite athletes (Sandbakk et al., 2016): In XC skiers, the total annual volume was on average 794 h with 646 h (81%) of LIT, 31 h (4%) of moderate-intensity training (MIT), 46 h (6%) of high-intensity training (HIT), and 71 h (9%) of strength and speed training. In NC athletes, the total annual volume was 836 h with 435 h (52%) of LIT (representing 87% of the XC ski training), 24 h (3%) of MIT, 39 h (5%) of HIT and 338 h (40%) of strength, speed, and jump training (Sandbakk et al., 2016). It appears than the XC ski training is “polarized” (LIT > 75% of total training, 81 vs. 87%) in both XC skiers and NC athletes, whereas the total volume of aerobic training was logically much higher in XC skiers (646 vs. 435 h).

It remains unknown if these differences in training content could influence the responses to LHTL in *XC skiers* and *NC athletes*. Therefore, in the present study, we aimed to analyze the effects on performance, maximal oxygen uptake, and HRV of LHTL (same altitude, same location) in two groups of XC skiers and NC athletes. We tested the hypothesis that during the LHTL period, a difference in low-intensity training volume (with the biggest amount of LIT in XC) and more strength and speed volume would induce less HRV perturbation, fewer fatigue states, and larger aerobic and performance improvement.

## Materials and Methods

### Subjects

Subjects were 20 elite Nordic-skiers, members of the *XC skiing* and *NC* French national teams. The characteristics of subjects were:

XC skiers group: 7 men (22.6 ± 2.8 year, 73.6 ± 5.9 kg, 179.9 ± 5.4 cm, $\dot{\text{V}}$O_2max_ 69.3 ± 3.6 mL⋅kg^-1^⋅min^-1^), and 5 women (22.8 ± 4.1 year, 55.6 ± 4.4 kg, 163.6 ± 5.2 cm, $\dot{\text{V}}$O_2max_ 58.9 ± 2.5 mL⋅kg^-1^⋅min^-1^).

NC group: 8 men (23.5 ± 4.5 year, 65.8 ± 3.1 kg, 176.3 ± 4.3 cm, $\dot{\text{V}}$O_2max_ 66.1 ± 3.2 mL.ckg^-1^⋅min^-1^).

All these athletes competed in European cup or World cup levels and some were medalists in World championships.

### Experimental Design

**FIGURE 1:**
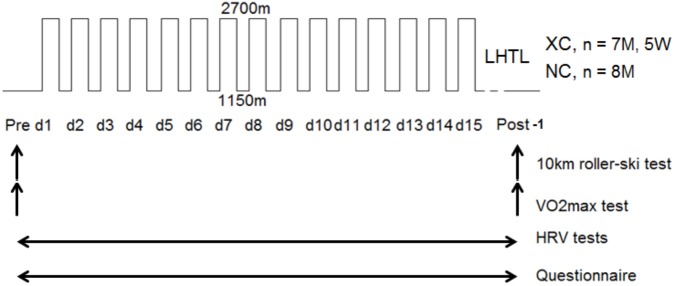
Experimental design. Cross-country skiers group (XC, men, *n* = 7, and women, *n* = 5), Nordic-combined group (NC, *n* = 8). The tests performed were a maximal *field* roller-skis test with blood lactate measurements 2 min after the end of exercise, a maximal oxygen uptake ($\dot{\text{V}}$O_2max_) on treadmill, a morning questionnaire upon awakening of fatigue, and an active Tilt test to measure the resting heart rate variability (HRV), and heart rate (HR). Vertical arrows indicate the day of the tests and horizontal arrows define the day-tests period.

The study design is shown in **Figure 1**. The study was approved by the local ethical committees (French National Conference of Research Ethics Committees; N°CPP EST I: 2014/33; Dijon, France). All experimental procedures conformed to the standards set by the Declaration of Helsinki (Harriss and Atkinson, 2015). All subjects provided written informed consent to participate in the study after having been informed in detail about the experimental procedure. Exclusion criteria for participation were any history of altitude-related sickness and health risks that could compromise the subject’s safety during training and/or hypoxic exposure. The study was performed at the French National Ski-Nordic Center which contains 11 hypoxic rooms and is located at an altitude of 1150 m. These hypoxic chambers were of medium size (15 ± 1 m^2^) and equipped with conventional beds. Two subjects were in each room and primarily spent time sleeping or resting between training sessions. The rooms were in normobaric hypoxic condition with FiO_2_ = 15.0% (2700 m) of simulated altitude and the training session were at 1150 m of real altitude. No athletes were under any medical treatment during the study.

### Training Performed

The intensity distribution was quantified depending of the time spent in heart rate (HR) zones delimitated by the correspondence between HR and the ventilatory thresholds. The TL was organized in four training zones as presented recently (Solli et al., 2017): *Intensity I* for endurance training at an intensity below VT1; *intensity II* for endurance training at an intensity between VT1 and VT2; *intensity III* for training at an intensity at VT2, interval-training at intensity above VT2, and competitions; and *intensity IV* for strength, speed training sessions, as well as jumping for the NC group.

VT1 corresponds to the first non-linear increases in $\dot{\text{V}}$CO_2_, and VT2 was determined as the first rise in the ventilatory equivalent of oxygen ($\dot{\text{V}}$E/$\dot{\text{V}}$O_2_) without a concurrent rise in the ventilatory equivalent of carbon dioxide ($\dot{\text{V}}$E/$\dot{\text{V}}$CO_2_) (Reinhard et al., 1979).

Training loads were quantified as described previously (Mujika et al., 1996) but slightly adapted to Nordic skiing. TL, expressed in arbitrary units (a.u.), was calculated by multiplying the training duration (in min) spent in each intensity zone by a coefficient (i.e., 1, 2, 4, and 8 for the zones I, II, III, and IV, respectively).

For the LHTL intervention, the intensity distributions planned by the national coaches and the main investigator were 80 - 5 - 5 - 10 for the training loads in intensity zones I, II, III, and IV for *XC* and 55 - 15 - 5 - 25 (including 15% jumping) for *NC*.

### Measurements

Measurements were performed 1 day before (Pre) and 1 day after (*Post*) LHTL intervention.

### Performance

A maximal roller-ski test was performed on an official FIS-accredited roller-ski asphalt track of the National French Ski-Nordic Center, with an equal alternation of up and down-hills, and flat parts. The women performed two laps, and the men three laps of 3.3 km in skating technique. Measurements were performed on each subjects’ own roller-skis and poles maintaining the same equipment use for each test.

### Lactate

The blood lactate concentration measurements ([La-], mmol.L^-1^) were performed 2 min after the end of the maximal roller-ski test with a Lactate Pro2^®^ analyzer (ARKRAY, Japan).

### Incremental Maximal Treadmill Test

The incremental running treadmill $\dot{\text{V}}$O_2max_ protocol used in the present study is routinely used for the medical follow-up of the French Nordic-skiers in the same laboratory. The test started at 8 km.h^-1^ and 2%-grade for 3 min, followed by 9 km.h^-1^ and 2%-grade for 3 min, 9 km.h^-1^ and 4%-grade for 3 min, 9 km.h^-1^ and 6%-grade, then 10 km.h^-1^ and 6%-grade. Every 3 min, the stage increased first by speed, then by grade until the second ventilatory threshold (VT2) was passed. VT2 assessment was made by visual inspection of graphs of time plotted against each relevant respiratory variable measured during testing. VT2 was determined as the first rise in the ventilatory equivalent of oxygen ($\dot{\text{V}}$E/$\dot{\text{V}}$O_2_) without a concurrent rise in the ventilatory equivalent of carbon dioxide ($\dot{\text{V}}$E/$\dot{\text{V}}$CO_2_) (Reinhard et al., 1979). After VT2, the protocol changed to 1 min stages at the same speed while increasing grade to a maximum of 16% until exhaustion. The same protocol was applied at Pre and Post tests. After VT2, the protocol changed to 1 min stages at the same speed while increasing grade to a maximum of 16% until exhaustion. The $\dot{\text{V}}$O_2max_ was determined by the highest 30 s average value. During all tests, HR was continuously monitored using a telemetry based HR monitor (Ambit3 Peak, Suunto^®^, Vantaa, Finland) and the maximum was obtained for further analysis. Oxygen (O_2_) and carbon dioxide (CO_2_) levels were continuously measured and monitored as breath-by-breath values in expired gas (Ultima Cardio 2^®^ gas exchange analysis system, MGC Diagnostics with Breezesuite software, Saint Paul, MN, United States). The flow meter and the gas analyser were calibrated prior to each test with a 3L volume calibration syringe (Hans Rudholph^®^, Medgraphics), and with a 5% CO_2_ and 12% O_2_ gas mixtures (Medgraphics).

### Questionnaire

The questionnaire of the French society of sport medicine (QSFMS) was completed each morning by all athletes. This questionnaire is used routinely by French national teams in various sports (rugby, swimming, Nordic-ski, triathlon…). The state of fatigue was registered when the score exceeded 20 negative items out of 54 (Benhaddad et al., 1999).

### Heart Rate Variability

The protocol of HRV tests is presented in detail in previous articles (Schmitt et al., 2013, 2015). Briefly, the HRV test consisted of a 15-min RR interval recording at rest with 8 min supine (SU) followed by 7 min standing (ST). The HRV recording was performed daily in the morning immediately after waking and voiding the urinary bladder. HRV analyses were performed on RR intervals between the 3rd and 8th min supine, and between the 9th and 14th min standing. Measurement of the interval duration between two R waves of the cardiac electrical activity were performed with a HR monitor (Ambit 3 Peak, Suunto^®^, Vantaa, Finland). Then the spectral power was calculated with the Fast Fourier Transform (FFT) using a software (Nevrokard^®^ HRV, Medistar, Ljubljana, Slovenia). The power of spectral density was measured by frequency bands in ms^2^.Hz^-1^ and the spectral power was expressed in ms^2^ (Task, 1996). The high frequency (HF) power band (0.15–0.40 Hz) reflects alteration of the parasympathetic influence on the heart and is related to the respiratory sinus arrhythmia (Pomeranz et al., 1985). While the low frequency (LF) power band (0.04–0.15 Hz) is also driven by parasympathetic tone, and presently considered responsible for carrying vagal resonances to either changes in vasomotor tone (often sympathetic) or in central modulation of sympathetic tone (Reyes del Paso et al., 2013). The spectral power in the LF power band has also been shown to be related to fluctuations of arterial blood pressure (Akselrod et al., 1981; Pomeranz et al., 1985) and to baroreflex activity (Goldstein et al., 2011). Both in supine (SU) and in standing (ST) positions, LF and HF were calculated in absolute spectral power units (ms^2^) and in normalized units (nu) with LF(nu) = LF/(LF + HF) × 100 and HF(nu) = HF/(HF + LF) × 100. The total spectral power (TP) was calculated by adding LF and HF. Additionally, the temporal analysis of the square root of the mean of the sum of the squares of differences between adjacent normal R-R intervals (RMSSD) was assessed.

### Statistical Analysis

In the two groups, XC and NC, data were tested for equality of variance (Fisher–Snedecor *F*-test) and for normality (Shapiro–Wilk test). When both conditions were met, a two-way repeated measures ANOVA [group (XC vs. NC) vs. measurement (Pre and Post)] was performed with pairwise multiple comparison procedures (*post hoc*, Tukey method). When either equality of variance or normality were not satisfied, variables were analyzed for each condition using a Friedman test for repeated measures to determine time effects using pairwise multiple comparison procedures (Bonferroni test). In this case, differences between the groups were tested using a Mann–Whitney rank sum test. The Pearson product moment correlation was applied to analyze the relationship between specific performance and lactate concentration changes. Null hypotheses were rejected at *P* < 0.05. Data are reported as mean and standard deviation (SD). These analyses were completed using SigmaStat 3.5 software (Systat Software, San Jose, CA, United States).

The statistical method used to distinguish the different HRV fatigue patterns in the NC group has been detailed previously (Schmitt et al., 2015, 2016). First, the analysis focuses on the difference in absolute values between no-fatigue and fatigue conditions, where a non-parametric Mann–Witney test was used to analyze the differences. Then, the relative difference between the mean of all no-fatigue and each fatigue state were calculated. The set of relative differences was submitted to a hierarchical clustering on principal components, which includes two steps. Firstly, a principal component analysis (PCA) was performed to disclose the organization of variables and to select the PCA dimensions which embed the main part of variance. Secondly, a hierarchical ascendant classification was performed according to the first dimensions of the PCA. This process limits the statistical signal to noise ratio and delineates clusters of individuals with similar characteristics. These analyses were conducted using the R-statistics software (V3.1.2) with the FactoMineR package.

## Results

### Training Load and Training Content

**FIGURE 2:**
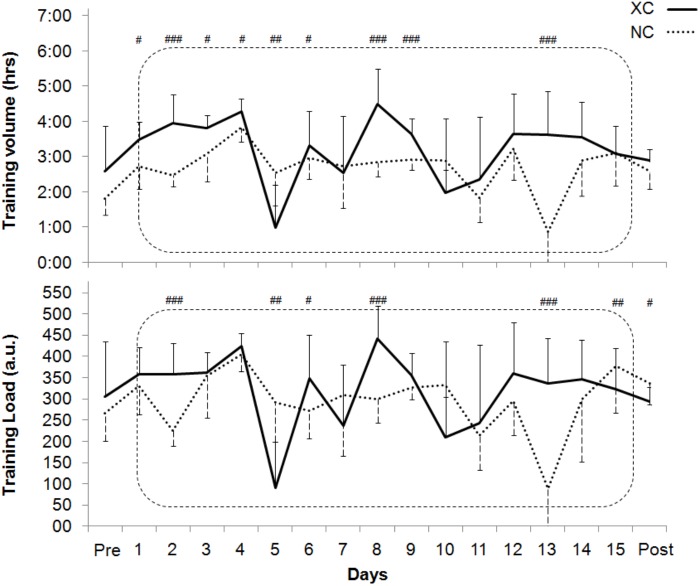
Volume of training (h), and training load (TL, in arbitrary unit, a.u.) performed during the Live High - Train Low study in the cross-country skiers (XC) and the Nordic-combined (NC) groups. With #*p* < 0.05, ##*p* < 0.01, ###*p* < 0.001 for differences between XC and NC.

In the 3 weeks preceding the LHTL intervention, both weekly TL and training volume were not different between XC (1557 ± 141 a.u., 16.0 ± 1.6 h) and NC (1733 ± 304 a.u., 16.5 ± 2.6 h). The training load and training volume of the LHTL period are shown in **Figure 2**. The intensity distribution for both groups are reported in **Table 1**.

**Table 1:** Content of the training performed in intensity zones with I for low-intensity training (LIT) below the first ventilatory threshold (VT1), II for moderate-intensity training (MIT) between VT1 and second ventilatory threshold (VT2), III for high-intensity training (HIT) upper than VT2, and IV for strength, speed training sessions, and jumping (only for Nordic-combined).

Groups	Intensity	Volume (h)	*P*	Total training volume (%)	*Cross-country training volume (%)*	*P*
XC	IV	7.1 ± 1.5		12.9		
	III	1.2 ± 0.9		2.2	2	
	II	1.9 ± 0.5		3.4	4	
	I	45.9 ± 6.4		81.6	94	
	Total	56.1 ± 6.5				
NC	IV	18.4 ± 2.7	<0.001	39.4		<0.001
	III	1.4 ± 0.2	0.2	3.0	5	0.05
	II	3.0 ± 0.4	<0.001	6.5	11	<0.001
	I	23.9 ± 2.8	<0.001	51.2	84	<0.001
	Total	46.9 ± 2.9	<0.001			


*XC (*n* = 12) for cross-country skiers, and NC (*n* = 8) for Nordic-combined. Data are expressed as mean ± SD. *P* for differences between XC and NC.*

### Performance and Lactates

Absolute values at Pre and Post are presented in **Table 2**.

**Table 2:** In cross-country (XC) skiers (*n* = 12) and Nordic-combined (NC) athletes (*n* = 8) groups respectively, field maximal roller-skiing test (min.s), Lactates (Mmoles.L^-1^) measured 2 min after the end of the roller-skiing test, maximal oxygen uptake ($\dot{\text{V}}$O_2max_, mL.mn^-1^.kg^-1^), and oxygen consumption at the second ventilatory threshold (VT2) ($\dot{\text{V}}$O_2V T2_, mL.mn^-1^.kg^-1^) at Pre and Post.

	XC		NC		*p*
			
	Pre	Post	Pre	Post	(Groups × Times)
Field roller-skiing test (min.s)	17.2 ± 2.0	17.1 ± 1.3 ^∗∗∗^	20.7 ± 0.4	21.0 ± 1.0 ^∗∗∗^	<0.001
Lactates (Mmoles.L^-1^)	12.1 ± 2.9	11.5 ± 2.8	18.2 ± 2.8	13.4 ± 3.2 ^∗∗∗^	<0.001
Treadmill test VO_2max_ (mL.min^-1^.Kg^-1^)	65.0 ± 6.2	67.3 ± 7.1 ^∗∗^	65.7 ± 3.1	68.1 ± 3.2 ^∗^	0.98
Treadmill test VO_2V T2_ (mL.min^-1^.Kg^-1^)	54.7 ± 4.9	58.7 ± 5.9 ^∗∗^	56.8 ± 4.2	58.0 ± 4.6	0.05


*Data are expressed as mean ± SD in absolute values. With ^∗^*p* < 0.05, ^∗∗^*p* < 0.01, ^∗∗∗^*p* < 0.001 for Pre vs. Post, and *p* for two way ANOVA (groups × times interaction).*

**FIGURE 3:**
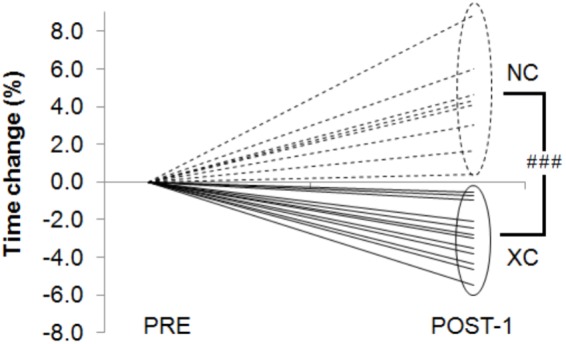
Time change (%) in the *field roller-skiing* test between Pre and *Post* in the cross-country skiers (XC) and the Nordic-combined (NC) groups. With ###*p* < 0.001 for differences between XC and NC.

The roller-ski performance was improved (-2.9 ± 1.6%, *p* < 0.001) in XC but decreased (4.1 ± 2.6%, *p* < 0.01) in NC (**Figure 3**). There was a significant group × measurement interaction (*p* < 0.001) (**Table 2**).

**FIGURE 4:**
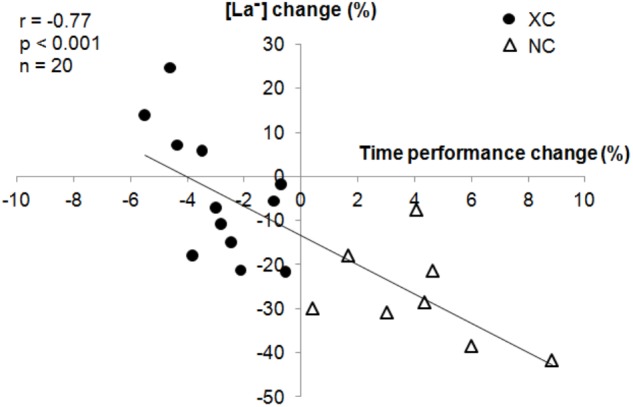
Correlation between changes (%) in blood lactate concentration ([La-]) and *maximal field roller-skiing* performance.

[La-] did not change (-4.1 ± 14.2%, *p* = 0.3) in XC but was decreased in NC (-27.0 ± 11.1%, *p* < 0.001). There was a significant group × measurement interaction (*p* < 0.001). A negative correlation (*r* = -0.77, *p* < 0.001) was found between the changes in [La-] and in roller-ski performance time (**Figure 4**).

### Incremental Maximal Treadmill Test

Absolute values at Pre and Post are presented in **Table 2**. From Pre to Post, maximal oxygen uptake ($\dot{\text{V}}$O_2max_) increased in both groups (XC: 65.0 ± 6.2 vs. 67.3 ± 7.1 mL.mn^-1^.kg^-1^, *p* = 0.01; NC: 65.7 ± 3.1 vs. 68.1 ± 3.2 mL.mn^-1^.kg^-1^, *p* = 0.002). The percent increase was not different between the two groups (3.7 ± 4.2%, *p* = 0.01 vs. 3.7 ± 2.2%, *p* = 0.002, for XC and NC, respectively). Oxygen consumption at VT2 ($\dot{\text{V}}$O_2V T2_) increased only in XC from Pre to Post-1 (7.3 ± 5.8%, *p* = 0.004 vs. 1.6 ± 4.3%, *p* = 0.31, for XC and NC, respectively).

### Questionnaire QSFMS

None of the XC or NC subjects declared a state of fatigue during the LHTL intervention and no difference in QSFMS scores were found between the groups (1.6 ± 1.5 a.u. in XC vs. 1.2 ± 1.4 a.u. in NC).

### Heart Rate Variability Parameters

The HRV parameters are displayed in **Table 3**. A trend was observed in difference of change between XC and NC (group × measurement interaction) in HF_SU_ (*p* = 0.05) and RMSSD_SU_ (*p* = 0.1).

**Table 3:** Heart rate and heart rate variability (HRV) parameters (in absolute and relative units) in supine (SU) and standing (ST) positions, 1 day before (Pre) and 1 day after (*Post*) live high – train low (LHTL) intervention.

		XC			NC		
			
		Pre	*Post*	*p*	Pre	*Post*	*p*
HR_SU_	bpm	*51.6* ± *5.7*	*54.9* ± *6.0*	*0.05*	46.8 ± 14.6	46.0 ± 20.8	0.13
	%	–	*6.9* ± *10.4*	*0.04*	–	6.0 ± 10.9	0.17
HR_ST_	bpm	77.2 ± 9.7	81.6 ± 8.8	0.23	*73.1* ± *21.5*	*72.4* ± *32.4*	*0.04*
	%	–	6.2 ± 12.5	0.09	–	*9.9* ± *11.6*	*0.05*
LF_SU_	ms^2^	2726 ± 2906	4294 ± 3485	0.17	3493 ± 2408	4479 ± 4164	0.89
	%	–	*188.0* ± *264.8*	*0.03*	–	70.0 ± 201.6	0.36
LF_ST_	ms^2^	5602 ± 3773	6585 ± 4174	0.41	6555 ± 4368	5932 ± 5027	0.79
	%	–	52.7 ± 126.8	0.18	–	-2.3 ± 71.0	0.93
HF_SU_	ms^2^	*4981* ± *3818*	*5151* ± *3299*	*0.77*	*6212* ± *4030*	*3562* ± *2638*	*0.17#*
	%	–	*30.6* ± *85.8*	*0.24*	–	*-8.3* ± *129.7*	*0.86#*
HF_ST_	ms^2^	1393 ± 2340	1244 ± 1703	0.64	819 ± 651	677 ± 515	0.25
	%	–	65.0 ± 171.6	0.22	–	-15.0 ± 36.0	0.28
LF + HF_SU_	ms^2^	7707 ± 5905	9444 ± 5616	0.09	9642 ± 5535	7951 ± 6241	0.43
	%	–	61.4 ± 101.4	0.06	–	16.8 ± 149.6	0.76
LF + HF_ST_	ms^2^	6995 ± 5627	7829 ± 5490	0.51	7333 ± 4615	6580 ± 5303	0.77
	%	–	47.5 ± 126.6	0.22	–	-3.4 ± 67.7	0.89
LF_SUnu_	n.u.	31.9 ± 17.3	44.8 ± 19.7	0.08	40.2 ± 14.9	47.1 ± 21.1	0.44
	%	–	68.1 ± 115.4	0.06	–	23.3 ± 58.5	0.29
LF_STnu_	n.u.	83.4 ± 12.2	86.6 ± 6.0	0.34	84.6 ± 23.0	77.5 ± 32.7	0.95
	%	–	5.4 ± 14.8	0.23	–	-0.1 ± 2.7	0.95
HF_SUnu_	n.u.	68.1 ± 17.3	55.2 ± 19.7	0.08	54.3 ± 17.5	40.6 ± 18.9	0.44
	%	–	-13.9 ± 35.5	0.20	–	-5.5 ± 39.2	0.70
HF_STnu_	n.u.	16.6 ± 12.2	13.3 ± 8.0	0.34	9.1 ± 3.3	8.4 ± 4.1	0.96
	%	–	17.5 ± 111.9	0.59	–	-0.1 ± 32.0	0.99
RMSSD_SU_	ms	108 ± 45	114 ± 34	0.49	115 ± 48	95 ± 51	0.31
	%	–	13.9 ± 38.2	0.23	–	-9.2 ± 42.5	0.56
RMSSD_ST_	ms	51 ± 25	42 ± 26	0.14	43 ± 15	34 ± 16	0.06
	%	–	-11.0 ± 38.3	0.34	–	-17.3 ± 22.4	0.06


*With heart rate (HR), low (LF) and high (HF) frequency spectral power, LF and HF in normalized units (LFnu, HFnu), and square root of the mean of the sum of the squares of differences between adjacent normal R-R interval (RMSSD). XC for cross-country skiers, and NC for Nordic-combined groups. Data are reported as mean and standard deviation (SD). With *p* < 0.05 for significant difference between Pre and *Post* and # (*p* < 0.05), in italic, for difference of change Pre vs. *Post* between XC and NC.*

**FIGURE 5:**
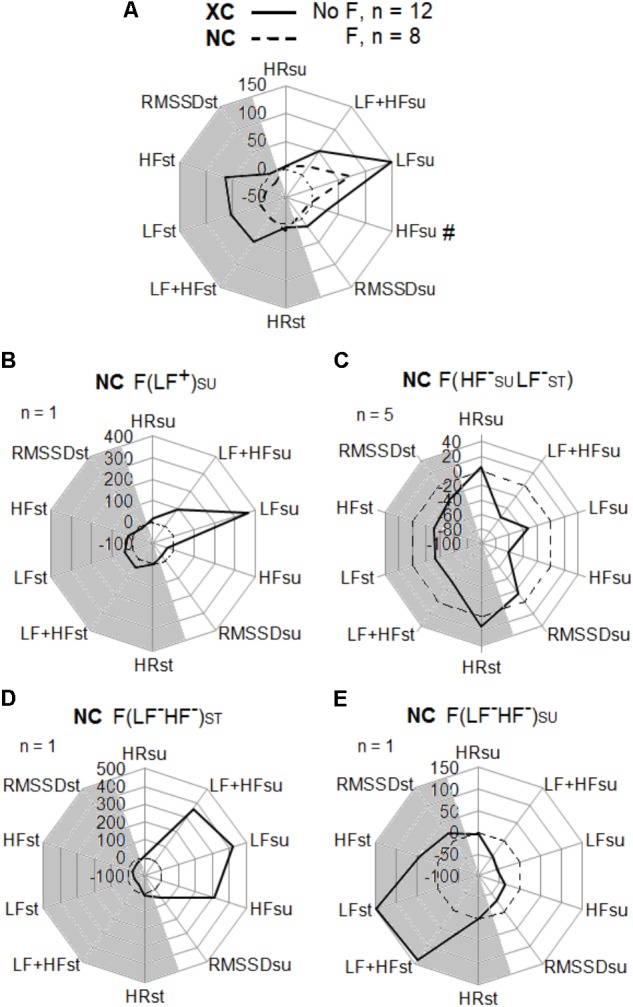
Heart rate variability, and heart rate change (%) between Pre and Post tests in the cross-country skiers (XC) and the Nordic-combined (NC) groups. HRV parameters in supine (SU) and standing (ST) positions with high (HF) and low (LF) spectral frequency powers, the addition of LF + HF, the temporal analysis of the square root of the mean of the sum of the squares of differences between adjacent normal R-R intervals (RMSSD), and the heart rate (HR). **(A)** XC (*n* = 12) with no fatigue (NF), and NC (*n* = 8) with fatigue (F). Fatigue (F) types were statistically sorted in NC: **(B)** F(LF^+^)_SU_ for fatigue with LF_SU_ increased, **(C)** F(HF^-^_SU_LF^-^_ST_) for fatigue with HF_SU_ and LF_ST_ decreased, **(D)** F(LF^-^HF^-^)_ST_ for fatigue with LF_ST_ and HF_ST_ decreased, **(E)** F(LF^-^HF^-^)_SU_ for fatigue with LF_SU_ and HF_SU_ decreased.

No fatigue states were observed in XC. In NC, four types of fatigue (F) were statistically sorted: F(LF_SU_^+^) for 1 subject, F(HF_SU_^-^ LF_ST_^-^) for 5 subjects, F(LF^-^HF^-^)_ST_, and F(LF^-^HF^-^)_SU_ for one subject each (**Figure 5**).

## Discussion

The main findings of this study were:

(1) LHTL induced specific endurance performance enhancement in XC but not in NC skiers, with a larger training volume in XC. The different total training intensity distribution between the two groups: in XC, 82-2-3-13 (i.e., ∼82% of TL < VT1) vs. in NC, 51-6-3-39 (i.e., ∼51% of TL < VT1), led to a specific (roller-skiing) performance enhancement in 100% of the XC subjects whereas it induced a performance decrease in all NC subjects.
(2) This was concomitant to different HRV responses between the groups since only NC athletes displayed fatigue states.

### Training Content and Intensity Distribution

The training content of the present athletes is similar to the values reported in Norwegian athletes (Sandbakk et al., 2016). Of interest is that both TL and training volume were higher in *XC skiers* than in *NC athletes* (**Figure 2** and **Table 1**). The *XC skiing* training performed by both groups is in accordance with the recommendation of the “polarized training” ‘75-5-20’ by Seiler (2010).

The training method named “polarized training” emphasizes the major influence of a high training volume performed at LIT (e.g., below the first ventilatory threshold, VT1) (Seiler and Kjerland, 2006). At the other range of the intensities spectrum, HIT is also a critical training component of all successful endurance athletes. It has been suggested (Seiler and Kjerland, 2006) that a ‘75-5-20’ training load (TL) distribution across the three intensity zones demarcated by VT1 and the second ventilatory threshold (VT2) would be optimal. Recently the intensity distribution ‘92-3-5’ was reported in the World’s most successful female *XC* skier over a 12-year period (Solli et al., 2017). Furthermore, “polarized” intensity distribution (‘80-12-8’) was more effective than “threshold training” (‘65-27-8’) in sub-elite runners over an 18-week period (10.4 km performance improvement of 157 vs. 121 s) (Esteve-Lanao et al., 2007). The XC training was polarized in NC athletes, but the high volume of strength-speed-jump training necessary for ski jumping performance possibly decreased the “polarization” influence (only 51% of TL at LIT). One may question if this low LIT volume would explain partly why LHTL was less effective in NC compared with XC.

The present results suggest that performing a high LIT volume is paramount during LHTL. In our view, this is particularly relevant and the “polarized” distribution is a prerequisite for inducing beneficial adaptations during altitude/hypoxic training period. It is known that training at LIT stimulates the parasympathetic branch of the neuro-vegetative system (Mourot et al., 2004). This type of training permits to avoid the onset of hypoxia-induced fatigue and is therefore effective for improving aerobic capacities and performance (Seiler et al., 2007; Schmitt et al., 2008). In NC, one may hypothesize that the pyramidal organization of the training intensities led to the observed misbalance in neuro-vegetative activity. The combination of a relative low stimulation effect of 51% of LIT on the parasympathetic activity and a high stress effect of 39% of strength-speed-jump training on the sympathetic activity could induce a fatigue effect and the subsequent decrease in specific performance reported in NC.

### Specific Performance and Lactate

The large decrease in [La-] observed only in NC (**Table 2**) suggests a large reduction in the glycolysis and glycogenosis maximal power of these subjects. The correlation (*r* = -0.77) between [La-] and time in the roller-ski test illustrates the influence of the glycolysis power in maximal endurance performance, as previously described (Urhausen and Kindermann, 2002). The [La-] decrement during maximal aerobic exercise has been shown as a marker of fatigue (Meeusen et al., 2013). It is important to observe that the XC subjects did not degrade in glycolysis maximal power despite a large volume at LIT whereas at the opposite of the NC athletes who performed less LIT. One possible explanation for this could come from the autonomous fatigue induced by the combination of hypoxic exposure and the high volume of strength, speed training sessions, and jumping in the NC subjects.

### Heart Rate Variability

Training loads was similar in XC and NC in the 3 weeks preceding LHTL and similar HR and HRV parameters were observed at Pre between XC and NC groups. Despite obvious physiological differences between these two groups, one could postulate that the fitness level was satisfying for both XC and NC at the beginning of LHTL.

The QSFMS questionnaire did not identify any fatigue during LHTL in both XC and NC. However, a HRV difference was observed in HF in supine position (**Table 3**), suggesting a lesser alteration of the neuro-vegetative balance in the XC group.

The current results are in accordance with those of Seiler et al. (2007), which demonstrated that highly trained athletes performing exercise for ≤120 min below the first ventilatory threshold (VT1) showed minimal disturbance in autonomic nervous system (ANS). Particularly in HRV temporal analysis with RMSSD and in spectral analysis with HF, these authors reported that the post-exercise parasympathetic reactivation/recovery was very fast. VT1 can be considered as a “binary” threshold for recovery in elite endurance athletes (Seiler et al., 2007).

In NC, four types of fatigue (F) were statistically determined (**Figure 5**). These types of fatigue were already described on Nordic-skiers (Schmitt et al., 2015) and in a swimming Olympic champion (Schmitt et al., 2016). For one subject, the fatigue type named F(LF_SU_^+^) was identified with increases in supine low frequency (LF_SU_) in spectral energy and in HR. This fatigue state represents a hypertonicity in sympathetic activity. The main type of fatigue F(HF_SU_^-^ LF_ST_^-^) was observed in five subjects and corresponds to a decrease in LF and high (HF) frequency observed in both supine and standing positions and accompanied by an increased HR. It presents a state of hypotonicity in the two branches (sympathetic and parasympathetic) of the neuro-vegetative system. For one subject, the fatigue type named F(LF^-^HF^-^)_ST_ was characterized by a decrease in standing LF accompanied by an increase in HR. Finally for one subject, the fatigue type named F(LF^-^HF^-^)_SU_ showed decreased LF and HF only in the supine position with increased HR and is related to a parasympathetic hypotonia (Seiler et al., 2007).

One important point of the present study is that these fatigue states were systematically associated to a decreased performance observed in all NC athletes but were not diagnosed by the questionnaire. This reinforces the practical usefulness of HRV that appears very sensitive to acute onset of fatigue in endurance athletes (Schmitt et al., 2015, 2016).

## Practical Applications

Several results of this study provide practical applications for sports coaches and researchers.

First, we confirm the effectiveness of LHTL in endurance athletes (as in the present elite *XC* skiers) as shown for 20 years in most of the existing literature (Bonetti and Hopkins, 2009; Millet et al., 2017) but also in excellent case studies (Solli et al., 2017). In addition, our results suggest that a large LIT volume is paramount during LHTL. We recommend having at least 80% of training time at LIT.

All the NC athletes reported a fatigue state at the end of LHTL. They had to perform a high percentage of strength and ski jumping training. This training in zone IV is focused on the development of force-speed and combined with a moderate volume of LIT could produce fatigue in LHTL. The present LHTL training content seems therefore less beneficial in NC athletes than in XC skiers.

Further research is needed to investigate if the addition/combination of recent hypoxic methods as resistance training in hypoxia or repeated sprint training in hypoxia (Girard et al., 2017) would be valuable in *NC athletes*.

## Limitations and Strengths

### Limitations

The main limitation concerns the design of the study. With a small number of subjects in NC (only eight subjects of the A and B French national teams), it was not statistically possible to split the group in two conditions; i.e., predominance of LIT vs. lower LIT content. Amendment of the training distribution is very difficult with elite athletes and to our knowledge there are only descriptive studies in this area with World-level athletes.

### Strengths

It is an applied study in ecological situation with “true” elite athlete; i.e., competing in European and World cups. Another strength is that the HRV fatigue states were related to the specific performance decrease. This reinforces the usefulness of monitoring different fatigue sub-categories as previously described by our research group (Schmitt et al., 2015, 2016).

## Conclusion

The present study showed that LHTL was beneficial in XC skiers but not in NC athletes. The training intensity distribution (i.e., ∼82% of LIT) of the XC group led to a specific (roller-ski) performance enhancement in 100% of the subjects whereas the lower LIT volume, and higher strength and speed training intensity distribution (i.e., ∼51% of LIT; ∼39% of strength and speed training) of the NC group induced an altered performance in all subjects, identified as overreached.

## Author Contributions

LS and GM designed the experimental protocol. LS, SW, NC, and GM performed the data collection and analysis. LS and GM wrote the manuscript. LS, SW, NC, and GM read and approved the final manuscript.

## Conflict of Interest Statement

The authors declare that the research was conducted in the absence of any commercial or financial relationships that could be construed as a potential conflict of interest.
